# Impact of rapid centrifugation on routine coagulation assays in South Africa

**DOI:** 10.4102/ajlm.v11i1.1901

**Published:** 2022-11-28

**Authors:** Reola Haripersadh, Dashini Pillay, Nadine Rapiti

**Affiliations:** 1 Department of Haematology, National Health Laboratory Services, Inkosi Albert Luthuli Central Hospital, Durban, South Africa; 2 Department of Haematology, School of Laboratory Medicine, University of KwaZulu-Natal, Durban, South Africa

**Keywords:** rapid centrifugation, coagulation assays, platelet-poor plasma, pre-analytic, Clinical and Laboratory Standards Institute guidelines, haemostasis

## Abstract

**Background:**

The recommendation for coagulation blood samples is to centrifuge at 4000 revolutions per minute (rpm) for 15 min to produce platelet-poor plasma before analysis. Rapid centrifugation, defined as centrifuging samples at higher speeds for shorter durations, could potentially reduce turn-around times (TAT), provided sample integrity is maintained.

**Objective:**

This study assessed the impact of rapid centrifugation on routine coagulation assay results.

**Methods:**

Blood samples were collected from volunteers at Inkosi Albert Luthuli Central Hospital and King Edward VIII Hospital, Durban, KwaZulu-Natal, South Africa, from September to November 2021. Samples were centrifuged using Method A, the current standard (4000 rpm/15 min), Method B (4000 rpm/10 min), Method C (5000 rpm/10 min) and Method D (5000 rpm/5 min). Platelet count, prothrombin time, activated partial thromboplastin time, thrombin time (TT), fibrinogen and D-dimer levels were analysed and results from Methods B, C and D compared to reference Method A.

**Results:**

Platelet-poor plasma was obtained from all samples (*n* = 60) using Methods A and B, and from 33/60 (55%) samples using Methods C and D. Differences between Method A and Methods C and D for normal prothrombin time, normal D-dimer and abnormal TT results were statistically significant. Prothrombin time results correlated strongly across all methods, while TT and D-dimer results correlated poorly. Activated partial thromboplastin time and fibrinogen results showed no significant differences across all methods.

**Conclusion:**

Rapid centrifugation at 4000 rpm/10 min (Method B) showed results consistent with the reference method. This method could potentially reduce the overall TAT for routine coagulation assays.

## Introduction

Laboratories are expected to provide accurate and reliable results within defined turn-around times (TATs) to facilitate the diagnosis, management and prognostication of patients.^1,2^ There are ongoing attempts to improve TATs as this will directly impact patient management.^3,4^ Coagulation assays are some of the most commonly ordered urgent and routine investigations.^1,2^ The recommended sample preparation method for coagulation testing is the centrifugation of whole blood to obtain platelet-poor plasma (PPP), defined as plasma with a platelet count of < 10 × 10^9^/L, which minimises interference by the platelet phospholipid surface, thereby preventing the activation of clotting factors.^5^ Obtaining PPP from whole blood requires the application of specific centrifugal forces over a given period.^6,7^ Minimal centrifugation times for routine coagulation assays vary across laboratories, often based on local observations.^8^ The Clinical and Laboratory Standards Institute guidelines recommend that whole blood collected in tri-sodium citrate tubes be centrifuged at 4000 revolutions per minute (rpm) for 15 min at room temperature to produce PPP.^9^ Strategies such as rapid centrifugation that are designed to reduce specimen processing times are being pursued as they could potentially reduce TAT in laboratories, eliminate the need for batch processing of samples and free up resources.^10^

As centrifugation time can be a bottleneck in coagulation testing, there is a need to determine the impact of rapid centrifugation on the laboratory workflow and TAT.^1,7,9^ Several studies have shown that centrifuging samples at higher speeds for shorter durations to obtain PPP has no significant effect on sample integrity and the accuracy of coagulation assay results.^11,12,13,14^

The primary goal of this study was to assess the impact of rapid centrifugation on the accuracy of routine coagulation test results.

## Methods

### Ethical considerations

The study was approved by the Biomedical Research Ethics Committee of the University of KwaZulu-Natal, South Africa (BREC/00002366/2021). Informed consent was obtained from each study participant and a unique study number was allocated to samples to ensure anonymity. The research data was stored electronically on password-protected devices and was only accessible by the researchers.

### Study design and setting

This study was conducted at the Inkosi Albert Luthuli Central Hospital and King Edward VIII Hospital, Durban, KwaZulu-Natal, South Africa, from September 2021 to November 2021. Patients older than 18 years were included in the study. Samples were excluded if there were insufficient blood volumes, or if clots, fibrin strands, or haemolysis were observed.

Sixty-two participants were included in the study (Table 1). Two samples were excluded from the data analysis due to tube breakage and one sample was treated as an outlier due to an abnormally high D-dimer result (35.2 mg/L) for Method A.^15^ From each participant, venous blood samples were collected into four 3.2% sodium citrate tubes (BD vacutainer 0.109 M/3.2 Citrate, Becton Dickinson, New Jersey, United States) labelled A, B, C and D, each representing one of the four centrifugation methods. Blood samples were collected and transported to the laboratory at room temperature (20 °C – 25 °C).

**TABLE 1:** Demographics and clinical diagnoses of study participants, Inkosi Albert Luthuli Central Hospital and King Edward VIII Hospital, Durban, KwaZulu-Natal, South Africa, September 2021 – November 2021.

Patient demographics and diagnosis	Participants	
	*N*	%
**Total number of participants†**	62	-
**Total number of samples included** ‡	60	-
**Sex**		
Male	33	55.00
Female	27	45.00
**Age (years)**		
18–30	21	35.00
31–40	15	25.00
41–50	8	13.33
51–60	8	13.33
61–70	8	13.33
**Diagnosis**		
Volunteers	12	20.00
Acute Leukaemia	8	13.00
Lymphoma	6	10.00
Coagulation disorders	8	13.00
Plasma cell dyscrasias	2	3.00
Myeloproliferative neoplasms	4	7.00
Other haematological disorders	10	17.00
Non-haematological disorders	7	12.00
Unknown	3	5.00

† , Samples from two participants were excluded due to the specimen being insufficient and tube breakage.

‡ , Of the 60 samples, one was treated as an outlier due to abnormally high D-dimer results (35.2 mg/L) using Method A.

### Centrifugation

Samples were centrifuged using two table-top Consul 22R (Ortoaltresa^®^, Madrid, Spain) instruments. The first instrument (Instrument 1) had a maximum rotor speed of 4000 rpm and required a 5 mL tube, while the second instrument (Instrument 2) had a maximum rotor speed of 9000 rpm and required a 50 mL tube (Table 2). A pilot experiment revealed that decanting the sample from the 5 mL citrate tube into the larger 50 mL tube before centrifugation resulted in a very thin supernatant plasma layer that was difficult to remove with a pipette. Placing the 5 mL citrate tube directly into the 50 mL tube resulted in the risk of tube breakage, therefore, cotton wool was used as a buffer between the two tubes. Samples were centrifuged within 4 h of phlebotomy.

**TABLE 2:** Speed and time specifications for centrifugation methods, Inkosi Albert Luthuli Central Hospital and King Edward VIII Hospital, Durban, KwaZulu-Natal, South Africa, September 2021 – November 2021.

Method	Speed (rpm)	Time (min)	Centrifugation instrument	Tube used
A (reference method)	4000	15	1	5 mL
B	4000	10	1	5 mL
C	5000	10	2	5 mL tube inside a 50 mL tube with cotton wool buffer
D	5000	5	2	5 mL tube inside a 50 mL tube with cotton wool buffer

min, minutes; mL, millilitres; rpm, revolutions per minute.

Method A was the Clinical and Laboratory Standards Institute-recommended reference method for centrifugation in the laboratory (4000 rpm for 15 min). Methods B (4000 rpm/10 min), C (5000 rpm/10 min) and D (5000 rpm/5 min) were compared to Method A.^9,16,17,18^ A literature search confirmed that centrifuging samples at 4000 rpm for 5 min did not produce PPP and therefore this method was excluded from the study.^19,20^ Samples for Methods A and B were centrifuged on Instrument 1, while samples for Methods C and D were centrifuged on Instrument 2. The supernatant plasma was transferred to an empty tube using a plastic pipette.^16,21^ All samples were tested on the Sysmex^®^ CS5100 (Siemens Healthcare GmbH, Marburg, Germany) automated coagulation analyser with reagents from Siemens Healthcare Products GmbH (Marburg, Germany). The reagents included Innovin^®^ (prothrombin time [PT] – clotting assay); Actin^®^ FSL (activated partial thromboplastin time [APTT] – clotting assay); Test Thrombin^®^ (thrombin time [TT] – clotting assay); Dade^®^ Thrombin Reagent (fibrinogen – clotting assay) and Innovance^®^ (D-dimer – immunoassay). The study samples were only analysed when the commercial controls were within the predetermined limits.^16,22^ An automated platelet count was performed on every sample using the Sysmex^®^ XN 3000 Automated Haematology analyser (Siemens Healthcare GmbH, Marburg, Germany) by flow cytometry based on principles of light scattering. This was done to assess the percentage of samples that produced a platelet count of < 10 × 10^9^/L in each of the four methods.

### Data analysis

Capturing of results, statistical tests and construction of Bland Altman plots were done using Microsoft^®^ Excel 2016 (Microsoft^®^, Redmond, Washington, United States). The EP Evaluator software version 8 (Informer Technologies Inc, Los Angeles, California, United States) and Stata version 17 (StataCorp^®^, College Station, Texas, United States) were used for statistical analysis. For descriptive statistics, numerical data were summarised as means, medians, standard deviations or percentages.^23,24^ The quality of the data was assessed using an acceptable correlation coefficient (*r*) value of > 0.975. The correlation coefficient (*r*) was used to assess the linear relationship between the reference method and Methods B, C and D.^25,26^ The paired student *t*-test was used to assess the statistical significance of differences between samples. The level of statistical significance was set at a *p*-value < 0.05. Percentage variations were compared with the current desirable quality specifications for bias and were derived from Westgard biological variation.^20,26^

## Results

Platelet-poor plasma was produced in all samples (*n* = 60) centrifuged at 4000 rpm for 15 and 10 min (Methods A and B) (Table 3). For Methods C and D, PPP was produced in 55% (33/60) of the samples, and 45% (27/60) of the samples had a platelet count between 11 and 90 × 10^9^/L.

**TABLE 3:** Platelet counts, prothrombin time, activated partial thromboplastin time, thrombin time, fibrinogen and D-dimer levels using four centrifugation methods, Inkosi Albert Luthuli Central Hospital and King Edward VIII Hospital, Durban, KwaZulu-Natal, South Africa, September 2021 – November 2021.

Test	Method A 4000 rpm/15 min				Method B 4000 rpm/10 min				Method C 5000 rpm/10 min				Method D 5000 rpm/5 min			
	No.	Mean	s.d.	Range	Mean	s.d.	Range	*p*-value	Mean	s.d.	Range	*p*-value	Mean	s.d.	Range	*p*-value
Platelet count (×10^9^/L)	60	1.18	1.23	0–5	1.77	1.63	0–8	0.02	9.35	7.66	0–38	< 0.001	10.53	12.65	0–90	< 0.001
**Prothrombin time**																
Normal (sec)	49	10.71	0.60	9.5–12.4	10.69	0.63	9.8–12.7	0.45	10.62	0.60	9.4–12.6	0.002	10.62	0.59	9.6–12.3	0.005
Abnormal (sec)	11	19.26	10.39	9.2–42.4	19.22	10.51	9.3–43.0	0.70	19.25	10.34	9.4–41.8	0.86	19.30	10.26	9.4–41.9	0.80
**Activated partial thromboplastin time**																
Normal (sec)	38	28.26	2.48	25–36.5	27.97	2.68	22.5–36.3	0.13	28.02	2.35	24.2–35.8	0.08	28.16	2.66	24.2–37	0.51
Abnormal (sec)	22	37.48	25.36	21.1–94.5	37.27	24.34	21–91.8	0.56	37.44	24.47	20.6–93.8	0.91	37.19	24.11	21.4–89.1	0.47
**Thrombin time**																
Normal (sec)	46	17.47	0.85	16–19	17.37	0.81	15.7–19	0.26	17.31	0.88	15.6–19.1	0.08	17.34	0.87	15.7–18.9	0.17
Abnormal (sec)	14	16.72	2.44	13.1–20.2	16.69	2.42	13.1–20.1	0.70	16.57	2.53	13.1–20.4	0.21	16.46	2.46	13.0–20.2	0.03
**Fibrinogen**																
Normal (g/L)	47	3.41	0.75	1.86–4.5	3.46	0.77	1.86–4.66	0.08	3.52	0.90	1.86–6.63	0.11	3.45	0.76	1.84–4.59	0.14
Abnormal (g/L)	13	5.28	2.15	1.21–8.24	5.18	2.38	1.20–8.9	0.63	5.44	2.32	1.22–8.9	0.06	5.37	2.31	1.21–8.9	0.26
D-dimer *																
Normal (mg/L)	19	0.20	0.02	0.19–0.25	0.27	0.26	0.19–1.34	0.29	0.21	0.03	0.19–0.27	0.04	0.21	0.03	0.19–0.28	0.02
Abnormal (mg/L)	40	1.5	2.05	0.26–9.06	1.49	2.05	0.27–8.9	0.89	1.44	2.06	0.23–8.93	0.52	1.47	2.10	0.22–9.04	0.74

min, minute; No., number; rpm, revolutions per minute; s.d., standard deviation; sec, seconds.

* , One sample was treated as an outlier due to abnormally high D-dimer results (35.2 mg/L) using Method A. *P*-values < 0.05 were considered statistically significant.

**FIGURE 1:**
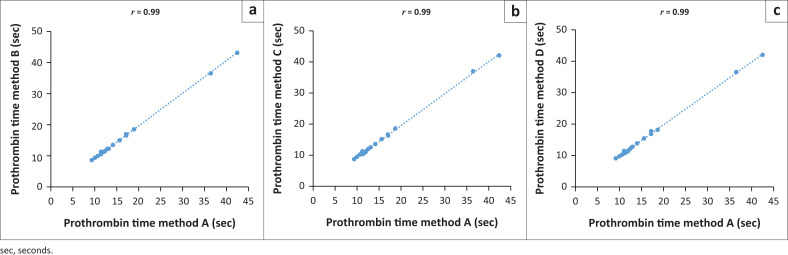
Comparison of prothrombin time results between the reference centrifugation method (Method A) and Methods B, C and D, Inkosi Albert Luthuli Central Hospital and King Edward VIII Hospital, Durban, KwaZulu-Natal, South Africa, September 2021 – November 2021. Linear regressions for: (a) Method A vs Method B, (b) Method A vs Method C, (c) Method A vs Method D.

Forty-nine (49/60; 82%) samples had a normal PT level (Method A: mean [seconds] = 10.71 s, median [seconds] = 10.6 s; Method B: mean = 10.69 s, median = 10.5 s; Method C: mean = 10.62 s, median = 10.5 s; Method D: mean = 10.62 s, median = 10.5 s). Eleven (11/60; 18%) samples had an abnormal prothrombin result (Method A: mean = 19.26 s, median = 15.6 s; Method B: mean = 19.22 s, median = 15.1 s; Method C: mean = 19.25 s, median = 15.5 s; Method D: mean = 19.30 s, median = 15.4 s). There were statistically significant differences for Method C (*p* = 0.002) and Method D (*p* = 0.005) in the normal PT group, with results being lower than the PT results for Methods A and B. Despite there being statistically significant differences in the normal PT results for Methods C and D, the PT results obtained using all three test methods correlated strongly with results obtained using the reference method (*r* ≥ 0.99) (Figure 1).

Thirty-eight (38/60; 63%) of the samples had normal APTT values (Method A: mean [seconds] = 28.26 s, median [seconds] = 27.9 s; Method B: mean = 27.97 s, median = 27.6 s; Method C: mean = 28.02 s, median = 27.7 s; Method D: mean = 28.16 s, median = 27.7 s). Twenty-two (22/60; 37%) samples had abnormal APTT results (Method A: mean = 37.48 s, median = 24.1 s; Method B: mean = 37.27 s, median = 24.4 s; Method C: mean 37.44 s, median = 24.3 s; Method D: mean = 37.19 s, median = 24.2 s). There were no statistically significant differences in the APTT results obtained using Method A versus Methods B, C and D; APTT results from the three processing methods strongly correlated with the results of the reference method (*r* > 0.975).

**FIGURE 2:**
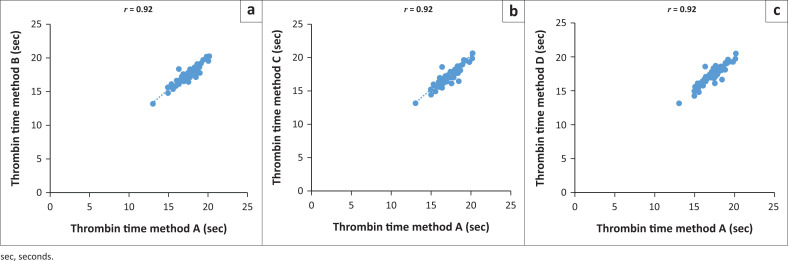
Comparison of thrombin time results between the reference centrifugation method (Method A) and Methods B, C and D, Inkosi Albert Luthuli Central Hospital and King Edward VIII Hospital, Durban, KwaZulu-Natal, South Africa, September 2021 – November 2021. Linear regressions for: (a) Method A vs Method B, (b) Method A vs Method C, (c) Method A vs Method D.

Forty-six (46/60; 77%) samples had normal TT levels (Method A: mean [seconds] = 17.47 s, median [seconds] = 17.5 s; Method B: mean = 16.69 s, median = 17.3 s; Method C: mean = 17.37 s, median = 17.3 s; Method D: mean = 17.34 s, median = 17.5 s). Fourteen (14/60; 23%) had abnormal TT levels (Method A: mean = 16.72 s, median = 15.6 s; Method B: mean = 16.69 s, median = 15.6 s; Method C: mean = 16.57 s, median = 15.5 s; Method D: mean = 16.46 s, median = 15.6 s). Abnormal TT levels were significantly lower among samples analysed using Method D compared to Methods A, B and C. All three methods correlated poorly (*r* = 0.92–0.93) with the reference method (Figure 2).

Forty-seven (47/60; 78%) samples had normal fibrinogen levels (Method A: mean [g/L] = 3.41, median [g/L] = 3.63; Method B: mean = 3.46, median = 3.61; Method C: mean = 3.52, median = 3.64; Method D: mean = 3.45, median = 3.57). Thirteen (13/60; 22%) samples had abnormal fibrinogen levels (Method A: mean = 5.28, median = 5.31; Method B: mean = 5.18, median = 5.43; Method C: mean = 5.44, median = 5.60; Method D: mean = 5.37, median = 5.40). The results of all three test methods correlated strongly with the reference method.

**FIGURE 3:**
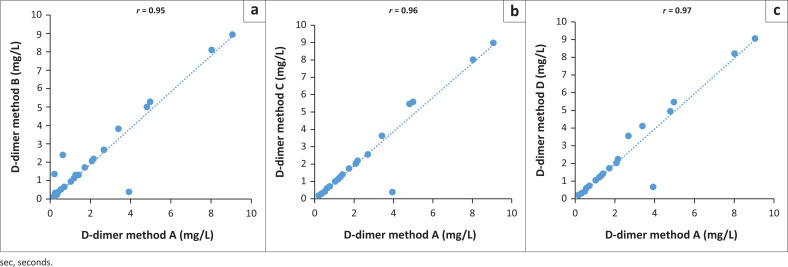
Comparisons of D-dimer results between the reference centrifugation (Method A) and Methods B, C and D, Inkosi Albert Luthuli Central Hospital and King Edward VIII Hospital, Durban, KwaZulu-Natal, South Africa, September 2021 – November 2021. Linear regressions for: (a) Method A vs Method B, (b) Method A vs Method C, (c) Method A vs Method D.

Nineteen (19/59; 32%) samples had normal D-dimer levels (Method A: mean [mg/L] = 0.20, median [mg/L] = 0.19; Method B: mean = 0.27, median = 0.19; Method C: mean = 0.21, median = 0.19; Method D: mean = 0.21, median = 0.20). Forty (40/59; 62%) samples had abnormal D-dimer levels (Method A: mean = 1.5, median = 0.58; Method B: mean = 1.49, median = 0.57; Method C: mean = 1.44, median = 0.56; Method D: mean = 1.47, median = 0.60). D-dimer levels were significantly lower for Methods C (*p* = 0.04) and D (*p* = 0.02) when compared to Method A. Method D correlated strongly with the reference method, while Methods B and C correlated poorly with the reference method (*r* < 0.975) (Figure 3). A single sample result for Method A (35.2 mg/L) had an extreme outlying value when compared with the results from Methods B, C and D (< 0.36 mg/L).

## Discussion

Our study found that centrifuging samples at 4000 rpm for 10 min yielded PPP in 100% of samples compared to centrifugation at 5000 rpm for 10 min and 5 min, which only yielded PPP in 55% of samples. The higher centrifugation speed caused an increase in platelet count in 45% of cases. This study has shown that Method B (4000 rpm/10 min) is superior to Methods C and D as it did not have a significant impact on the coagulation assay results. Hence, Method B could be an alternate method of processing samples for coagulation tests.

A study by Barnes et al showed that 10 min was the minimum centrifugation time required to consistently meet the recommendations for PPP.^27^ This differed from a previous study by Sultan et al., which reported that the majority of the 46 samples tested produced PPP when centrifuged at 5000 rpm for 5 min.^12^ Although PPP is recommended for coagulation testing, studies suggest that coagulation test results are not affected by platelet counts of > 10 × 10^9^/L and that samples with platelet counts of up to 199 × 10^9^/L produce similar results to samples with PPP.^28^ In our study, platelet counts differed significantly between the samples centrifuged at 4000 rpm (Method A and B) and those processed at 5000 rpm (Method C and D); however, there were no statistically significant differences between Method A and Methods B, C and D in results for the APTT and fibrinogen assays, as well as for the abnormal PT, normal TT and abnormal D-dimer assays. This is similar to the findings of previous studies conducted in 2002 and 2011.^19,27^

Statistically significant differences were observed in the normal PT and normal D-dimer assay results when centrifuged at 5000 rpm compared to the reference method. Abnormal TT levels were significantly lower when measured with Method D compared to Method A; however, this was not the case for Method C which was also a 5000-rpm method. Although the results for normal PT were found to be statistically significantly different between the reference method and Methods C and D, the differences in the actual mean, median and standard deviation values between the groups were minimal. When combined, the normal and abnormal PT results showed a good correlation between the different methods (*r* = 0.99) and these results are similar to those discovered in a previous study by Azlin et al.^19^

There were statistically significant differences in the abnormal TT (Method D) and normal D-dimer values (Methods C and D) when compared to the reference method; however, there was minimal variation in the mean, standard deviation and median values. Furthermore, the TT results showed poor correlation (*r* = 0.92–0.93) for all three methods when compared to the reference method. These findings suggest that the variation of the raw data may not be clinically significant as the clinical management of patients in our setting would not be altered.

The APTT assay is usually more sensitive to platelet contamination than the PT assay.^19^ This was not observed in our study as the results of the APTT and fibrinogen assays showed no significant differences and correlated strongly between the test methods and the reference method.

A single D-dimer result from Method A had an outlying value when compared with the results from Methods B, C and D. It was confirmed that the sample was collected following standard practice guidelines, labelled correctly and processed using the standard operating procedure of the laboratory.^29^ Processing errors can occur during the pre-analytical phase^8,30^; however, a transcription error was excluded in this case. Owing to limited sample availability and repeat sampling not being possible, Method A could not be rerun.^30,31^

Our study results encourage further research on rapid centrifugation of coagulation samples to verify the reliability of the results and explore the potential benefits it could have in a clinical laboratory setting. These findings could have practical applications and serve as a basis for additional research to establish local centrifugation protocols in laboratories.^19^

### Limitations

The results of this study (sample size = 60) need to be validated with a larger case-control study. A larger number of healthy individuals (controls) should be included. The PT, TT and fibrinogen assays had low abnormal sample numbers ranging from 20% to 30% and the TT results did not reflect extreme abnormal ranges. Participants were recruited on a voluntary basis; therefore, some assays showed a bias in the normal-to-abnormal ratios.

### Conclusion

This study demonstrates that Method B is superior to Methods C and D as it produced results that were most consistent with those obtained using the reference method. Methods C and D produced statistically significant differences in results for the PT, TT and D-dimer assays. We show that the centrifugation of whole blood samples in 5 mL citrate tubes at 4000 rpm for 10 min is suitable for routine coagulation testing. This rapid centrifugation method provides consistent and reliable results and could potentially reduce the overall TAT. These findings may assist experts in revising the current recommendations for the centrifugation of coagulation samples.
